# Opportunities and Scope for Botanical Extracts and Products for the Management of Fall Armyworm (*Spodoptera frugiperda*) for Smallholders in Africa

**DOI:** 10.3390/plants9020207

**Published:** 2020-02-06

**Authors:** Naomi B. Rioba, Philip C. Stevenson

**Affiliations:** 1 School of Agriculture and Biotechnology, University of Kabianga, Kericho P.O. Box 2030-20200, Kenya; naomirioba@kabianga.ac.ke; 2 Natural Resources Institute, University of Greenwich, Chatham Maritime, Kent ME4 4TB, UK; p.c.stevenson@gre.ac.uk; 3 Jodrell Laboratory, Royal Botanic Gardens, Kew, Richmond, Surrey TW9 3AB, UK

**Keywords:** biopesticides, botanicals, corn, insects, pests, prospects

## Abstract

Fall Armyworm (FAW) (*Spodoptera frugiperda*) is a polyphagous and highly destructive pest of many crops. It was recently introduced into Africa and now represents a serious threat to food security, particularly because of yield losses in maize, which is the staple food for the majority of small-scale farmers in Africa. The pest has also led to increased production costs, and threatens trade because of quarantines imposed on produce from the affected countries. There is limited specific knowledge on its management among smallholders since it is such a new pest in Africa. Some synthetic insecticides have been shown to be effective in controlling FAW, but in addition to the economic, health and environmental challenges of pesticide use insecticide resistance is highly prevalent owing to years of FAW management in the Americas. Therefore, there is a need for the development and use of alternatives for the management of FAW. These include plant-derived pesticides. Here we review the efficacy and potential of 69 plant species, which have been evaluated against FAW, and identify opportunities for use among small-scale maize farmers with a focus on how pesticidal plants might be adopted in Africa for management of FAW. The biological activities were diverse and included insecticidal, insectistatic (causing increased larval duration), larvicidal, reduced growth and acute toxicity (resulting in adverse effects within a short time after exposure). While most of these studies have been conducted on American plant taxa many South American plants are now cosmopolitan weeds so these studies are relevant to the African context.

## 1. Introduction

Fall Armyworm (FAW) (*Spodoptera frugiperda* Hurst) (Lepidoptera: Noctuidae) is a highly polyphagous pest having been reported on more than 80 species in 23 families [1] including cotton (*Gossypium hirsutum* L.) (Malvales: Malvaceae), corn (*Zea mays* L.) (Cyperales: Poaceae) and many other grass crops [2]. Originally native and restricted to the Americas, FAW was recorded for the first time in Africa in 2016 [3] and now it has spread to over 30 countries in Africa. 

These invasive populations are now well established and causing severe destruction to important crops that underpin the livelihoods of many farmers across Africa [4], due to the variety of host plants and the favorable environment and climate. The pest has characteristics that means it presents a wider-reaching threat to Africa [3]. For example, in comparison with the African armyworm *(Spodoptera exempta)*, FAW larvae have unique mouthparts with notched cutting edges, enabling it to feed on flora that are rich in silica content. More so, the older larvae feed on the younger ones and can dominate the competitors of the same species and others of different species within the same genus hence ensuring its survival [5]. FAW has raised greater concern among farmers than related African *Spodoptera* species because it causes especially severe damage to maize, feeding on virtually all parts of the plant leading to considerable damage, and sometimes results in total crop failure [6].

Sustainable approaches to managing this new African pest should ideally be integrated, tailored and appropriate for smallholders with mixed cropping farming systems and reduced input costs. While the use of chemical pesticides dominates existing approaches [7], several alternative control options exist and are being considered including resistant varieties [8,9,10], biological control [11,12], crop management practices [13,14], plant diversity [14], and mechanical methods [15]. However, none of these methods has yet delivered a viable option for effective control of FAW, hence the search for alternative approaches including those from plant extracts and their products. Some pesticidal plants and botanical insecticides are effective and their use could reduce reliance on synthetic insecticides since they have lower non-target impacts and could even boost growth [16,17,18,19]. Here we review existing research on plant extracts that have been evaluated for the management of FAW with the aim of identifying those with potential for use by small-scale farmers in Africa, or informing approaches to identifying and evaluating untested native African plant taxa since pesticidal plants are already used as crudely produced products among smallholder farming communities in Africa with notable success [20,21,22]. While one recent study has specifically sought to evaluate African plant taxa for activity against FAW [23], most of the studies reviewed here have been conducted on South American taxa but many of these species are now cosmopolitan weeds so are relevant to the African context. For example, *Ageratum conyzoides* L. is a widely used plant for a multitude of uses in Africa including pest control but originates from South America where it has been evaluated for efficacy against FAW [24,25]. Similarly, *Dysphania* (syn. *Chenopodium*) *ambrosioides* (L.) Mosyakin & Clemants, has been shown to be biologically active vs FAW [26,27] but is also considered for use in Africa [28], while species such as *Corymbia* (syn. *Eucalyptus*) *citriodora* (Hook.) K.D. Hill & L.A.S.Johnson are widespread in both Africa and America but non-native and have been evaluated for activity against FAW [29]. 

## 2. Opportunities and Potential of Botanical Extracts and Products

Interest in using plant extracts for pest control is increasing since these can: 1) reduce the cost of production of the crop, 2) reduce the environmental damage and non-target effects, and 3) reduce dependence on synthetic insecticides [30,31]. There are many researchers studying insecticidal plants for the control of FAW with several reporting promising results, although many do not since they do not establish the chemical basis of activity or store any reference specimens [32]. Some of the pesticidal plant species that have been shown to be effective in the management of FAW are presented in Table 1

FAW larvae ingesting maize leaves treated with the essential oil of *Ageratum conyzoides* were killed with 70% mortality caused at the concentration of 0.5%. The essential oil contained precocene as the major active component (87%) [24]. This finding is highly relevant to the African context where this plant grows widely on farmland. This means that it is easily available to farmers. It has already been used to control lepidopteran and other pests by some small holders in Africa. It has been shown to have reduced non-target effects on natural enemies of pests [85].

Ruta graveolens, Cymbopogon citratus, Zingiber officinale, Malva sylvestris, Petiveria alliaceae, Bacharis genisterlloides and Artemisia verlotorum were also shown to cause mortality for caterpillars of FAW [36], but active components in these plants were not identified, meaning that the work has limited value in the efforts to develop new approaches for FAW control unless more research is conducted to identify the active compounds that are responsible for the biological activity. 

The essential oil of *Cymbopogon flexuosus* was reported to be lethal to FAW (LC_50_ = 1.35 mg ml^−1^) when supplemented in to an artificial diet at 2.25, 2.5 and 4 mg ml^−1^ concentrations and 18.85 h median lethal time (LT_50_). The insecticidal activity of citral was not significantly different to the essential oil, suggesting that citral, a compound of this essential oil caused insecticidal effects of the *Cymbopogon flexuosus* essential oil to FAW [80].

*Moringa* oil induced a lower feeding ratio expressed as the ratio of consumed area of treated leaf discs to consumed area of untreated (control) leaf discs and highest total corrected mortality percentage of FAW. This study concluded that at 10% concentration, *Moringa* oils can be used as a botanic insecticide in the management of FAW. Saponifiable components of the *Moringa* oils comprised of oleic acid (74.2%) and palmitic acid (7.16%). However, the LC_50_ of moringa oil, unsaponifiable and saponifiable matters were 1.9%, 3.4% and 7.6% respectively, indicating that saponifiable matter was less effective against FAW larvae [77]. This therefore, means that there is no need for separation and identification of the moringa oil components for application in FAW control. Farmers should be advised to apply whole *Moringa* oil to benefit from the synergistic effects of the components therein.

Linalool showed potential in controlling FAW through non-preference, knockdown and toxicity effects on FAW larvae [56,57]. More than 80% of the essential oil of *Ocimum basilicum* consisted of linalool suggesting that this is the main active component [86]. More recently this species has been evaluated against FAW in Africa as part of a study focused on plants that were either native or widely grown in Malawi [23]. Another species investigated in this study included *Tephrosia vogelii*, a rotenoid producing and widely used species for pest control in Africa but this was not active suggesting a level of tolerance in FAW to the insecticidal rotenoids occurring in this species [23,87]. Another South American plant which grows widely as an invasive weed in Africa where it has been shown to have biological activity against insects [88] and used widely as a pesticide is *Tithonia diversifolia* but again this species was not active [23]. The most promising plant species based on their low mammalian toxicity, abundance and bioactivity against FAW identified through this work were *Lippia javanica, Ocimum basilicum* and *Cymbopogon citratus* which showed various activities including anti-feedancy and increased mortality. These three species are consumed as spices and teas so are far safer than synthetics [23]. *C. citratus* has also been shown in studies elsewhere to be effective against FAW. For example, it was been reported that sub-lethal doses of citronella oil altered the biochemical profile of FAW larvae causing damage to their reproductive histophysiology and resulted in diminished reproduction or reproductive failure [42]. The citronella-treated midgut of FAW larvae displayed modifications to the epithelium such as increased periodic acid-Schiff positive granules, columnar cell extrusion, cytoplasmic protrusions and pyknotic nuclei [42]. This study showed further that there was an increase in regenerative cells, which aided successive renewal of the epithelium. Trophocytes which are the main cell type of the fat body, once exposed to citronella, had reduced amounts of proteins, glycogen, and lipids. The fat bodies also showed distended vacuoles and mitotic bodies. This implies that citronella oil acts by causing changes in the morphology of the midgut and reducing stored resources in the fat body, limiting insect reproduction and survival.

FAW larvae feeding activity was reduced when treated with 1% and 10% methanolic extract of *Melia azedarach* seed. Other effects were slowed caterpillar growth due to ingestion of toxic substances present in *M. azedarach* [52,61], extended pupation time, small pupae and deformed moths. While native to Indomalaya and Australasia *Melia azaderach* grows widely in South America and Africa as well, so this study is highly relevant to the African context although there is some concern about the toxicity of the plant [89]. 

The ethanolic extract of Poinsettia *(Euphorbia pulcherima*) leaves obtained during the vegetative and reproductive phase was evaluated against FAW. The extracts were fed to the FAW larvae after mixing with artificial diet. Administered at 0.5 and 1% concentrations, the extracts increased the larval period, reduced larval and pupae weight as well as egg viability and resulted in greater larval mortality. It was further noted that the extract prepared from leaves that were in the reproductive phase of the plant effectively reduced the FAW population. Cold aqueous extract of *E. pulcherrima* also resulted in 58.5% mortality of FAW [53; 54] affecting *Neonotonia wightii* (perennial soybean).

Trials were done on the bioactivity of aqueous plant extracts of *Calotropis procera*, *Jatropha curcas*, *Cymbopogon nardus* (citronella), *Zyzyphus joazeiro*, ‘noni’, *Morinda citrofolia*, *Magonia pubescens* and *Annona squamosa* and showed that the consumption of leaves impregnated with different plant extracts increased larval mortality and significantly decreased pupal weight. The *Annona squamosa* treatment had the most effective insecticide activity against FAW. However, no identification of the phytochemicals responsible for these activities was done making the exploitation of these data difficult [33].

Methanolic extracts of leaves, bark and fruit peel of *Copaifera langsdorffii* resulted in low FAW egg viability [79]. Findings of this study showed that the methanolic extracts from leaves and fruit peel added to the artificial diet of 2nd instar FAW had several effects including reduced larval growth, long development duration, lower fertility and fecundity of adults as well as augmented mortality. The aeropylar and micropylar regions of the eggs had abnormalities. The insect feces were high in protein as reflected by repressed trypsin activity in the in vitro test. They suggested that *C*. *langsdorffii* presented the greatest potential for use as alternative bioinsecticide for control and management of FAW.

The effects of aqueous extracts of *Talisia esculenta* and *Sapindus saponaria* on the FAW at 8 and 14 days of development led to increased larval mortality at 63.15% and 26.71% for *S. saponaria* and *T. esculenta*, respectively [61]. The extract of *T*. *saponaria* was the most promising for the control of FAW. This might be because their seeds high in fat content, yielding a similarly fatty extract with adjuvant capacity thus facilitating fixing and distribution of the extract on the leaves of maize hence increasing the insecticide action. There remains, however, the need to determine the insecticidal compounds in the plants on whose basis new natural insecticidal products could be produced or improvements to the extraction could be made. 

A study under laboratory conditions and conducted on the biological activity of boldus (*Peumus boldus* Molina) water extract against FAW and *Helicoverpa zea* (Boddie) (Lepidoptera: Noctuidae) [76] showed that FAW was the most susceptible with 75% mortality at seven days when exposed to 8% w/w of *P*. *boldus* extract and had an LC_50_ value of 2.31 mL kg^−1^. Again, no chemistry was undertaken making the usefulness of this data questionable. 

The bioactivity of *Ipomoea murucoides* methanolic extracts and fractions on FAW were evaluated by incorporating the extracts into a meridic diet at concentration of 2 mg mL^−1^. These were then fed to FAW larvae (1st instars) [48]. After seven days, crude leaf extracts caused up to 46.16% mortality (leaf extract LC_50 =_ 2.692 mg mL^−1^). Other effects were reduced larval weight, increased pupation time and in the time to attain adulthood. No influence was noted for the number of eggs. Despite the fact that the partly purified fraction caused no toxicity to FAW, the greatest effect was on reduced larval weight, augmented pupation time and time to attain adulthood with an influence on number of eggs.

An earlier study on a methanolic extracts of *I. murucoides* calli [49] reported that it induced a higher (95%) neonate larvae mortality than was reported by [48]. This difference was explained by the fact that in [48], the leaf extracts contained a large amount of chlorophyll (that is lacking in calli) which masked the compounds and therefore inhibited their activity. 

A study reported in [37] investigated how tocotrienols and hydroquinones from *Roldana barba-johanis* affected the growth of insects. The major compounds obtained from the aerial parts methanol extract were sargachromenol, sargahydroquinoic acid and sargaquinoic acid. These compounds and their associated methylated and acetylated derivatives exhibited insect growth regulatory and insecticidal activities against the FAW. The most biologically active phytochemicals were sargachromenol, sargahydroquinoic acid and sargaquinoic acid in the order of abundance. These compounds and the acetylated form of this mixture resulted in negligible effects. When used at 5.0 and 20.0 ppm in diets, they caused substantial inhibitory effects on FAW larvae with insecticidal activity ranging between 20 and 35 ppm. 

*Eucalyptus citriodora* Hook (Myrtaceae) contained eucalyptin in methanol extract of leaves along with naringenin, chrysin, apigenin, quercetin, and luteolin, oleanolic acid, ursolic acid, betulinic acid and composite mixtures of flavonoids and triterpenes that were not identified [29]. These compounds exhibited insecticidal and insect growth regulatory and antifeedant activities, against FAW and the Yellow Mealworm (*Tenebrio molitor*) (Coleoptera:Tenebrionidae).

The sublethal effects of the essential oils of *Foeniculum vulgare*, *Ocimum gratissimum* and *Eucalyptus staigeriana* on FAW have been reported [34]. The essential oils caused reduced larval and pupal weights, increased larval and pupation periods, reduced oviposition period and adult survival although there were variations in effects. The essential oil of *O*. *gratissimum* had the greatest effects across the tested doses. These insecticidal effects could have been as a result of essential oil components like limonene, geranial, (*E*)-anethole, eugenol and *α*-pinene in the essential oils. This provides an opportunity for researchers to explore other plants with these compounds with the aim of incorporating them in the pool of plants that provide promising outcomes for managing FAW in Africa.

Methanol extracts of *Yucca periculosa* bark yielded 4, 4′-dihydroxystilbene, resveratrol and 3, 3′, 5, 5′-tetrahydroxy-4-methoxystilbene. These compounds showed growth regulatory effects against the FAW. The most active compound was 3, 3′, 5, 5′-tetrahydroxy-4-methoxystilbene which was active at 3 µg g^−1^ in diets [35]. However, the utilization of *Y*. *periculosa* (Agavaceae) is limited due to its local use as a source of firewood. In addition, the leaves of this plant are used for making handicrafts while the flowers are utilized as food. At 25.0 ppm concentration, the methoxy stilbene and methanolic extract of *Y*. *periculosa* caused 100% mortality of larvae. Most importantly, the methoxy stilbene and methanolic extract of *Y*. *periculosa* proved to be more active than gedunin and the methanolic extract of *Cedrela salvadorensis* with LC_50_ values of 5.4 ppm and 7.18 ppm, respectively. They also indicated that there was a decrease in the percentage of larvae attaining pupation across treatments as compared to the control. Survival of the pupae was reduced to 0.05 at 25 and 50 ppm for the methoxy stilbene and methanolic extract, respectively. The percentage of adult emergence showed further impacts at the pupal stage with resveratrol, the methoxy stilbene, methanolic extract of *Y*. *periculosa*, gedunin and methanolic extract of *C. salvadorensis* with 0.0%, 27.0%, 18.0%, 13.0%, and 8.0% of emergence, respectively at 25, 10, 10, 25 and 25 ppm. The methoxy stilbene and the methanolic extract of *Y*. *periculosa* with Relative Growth Index (RGI) values of 0.25 and 0.45 at 10 and 15 ppm gave the greatest outcome. The effects of resveratrol, the methoxy stilbene and methanolic extract of *Y*. *periculosa* did not differ from that of gedunin but had greater potency than the methanolic extract of *C. salvadorensis* [64). This finding presents these plants as having potential for further development for use against FAW.

The aerial portions of *Gutierrezia microcephala* yielded four oxyflavones, which were tested for activity against neonate larvae of FAW [38]. The flavone, a clerodane, its methyl ester, methanolic and n-hexane extracts caused a major delay in the time taken to attain pupation and adult emergence. Severe toxicity against FAW adults and insect growth inhibition were also reported [38].

*Maytenus disticha* aerial parts and *Maytenus boaria* seeds were evaluated to determine their effects on the FAW [47]. Several β-dihydroagarofurans were isolated including 9-benzoyloxy-1,2,6,8,15-pentaacetoxy-dihydro-β-agarofuran-(1) and 9-furanoxy-1,6,8-triacetoxy-dihidro-β-agarofuran and their insecticidal activities compared to ethanol extracts from *A. indica* and *M. azedarach* [65]. There was a 58% and 100% growth inhibition at 16 and 80 ppm, respectively. This suggested that agarofurans and MeOH and hexane/EtOAc extracts from *M*. *disticha* and *M. boaria*, respectively, have potential for use as a biopesticide against FAW. 

Extracts of *A. indica* and *M. azedarach* caused significant larval deaths, slowed the growth rate of larva and lengthened pupation time. The influence of 9-furanoxy-1,6,8-triacetoxy-dihidro-*β*-agarofuran and hexane/EtOAc extract on FAW was comparable to that of limonoids such as gedunin and cedrelone [90]. The action of these compounds was comparable to toosendanin, which is a commercially available biopesticide, suggesting that there is potential for researchers to harness these plants and produce products that can assist in controlling FAW.

*Ricinus communis* has been identified as a potentially important pesticidal plant owing to its insecticidal properties. Some fatty acids obtained from the aqueous extracts of caster plant have shown insecticidal and insectistatic activity against FAW. For example, linoleic acid, palmitic acid and stearic acid show biological activity against FAW [46] while linolenic acid was reported to have insecticidal and insectistatic activities against FAW [51]. 

Castor oil and vicinine which can be extracted from seeds or leaves of *R. communis* were active against FAW, however, the seed extract was more potent [50]. The two test substances were associated with the effects observed for FAW. The half maximum larvae viability concentration (LVC_50_) was 0.38 × 103 ppm for the vicinine, 0.75 × 103 ppm for methanol extract of seeds, 1.97 × 103 ppm for ethyl acetate seed extract, 2.69 × 103 ppm for castor oil, 4.83 × 103 ppm for a methanol extract of leaves and 10.01 × 103 ppm for a hexane extract of leaves. Bioactivity in castor plants is particularly relevant to the African context as this plant is cosmopolitan and grows abundantly adjacent to farmland in many parts of Africa. 

*Trichilia pallida* leaf and branch extracts when applied at very low concentrations of ≤ 0.0008% were shown to have no effects on eggs and larvae of FAW [63]. Although less diverse species of the genus *Trichilia* also occur in Africa such as *T. emetica* indicating the potential for using knowledge from American studies to inform the use of African species. 

Insecticidal activity of *Salvia* spp. has also been reported on FAW and *Spodoptera littoralis* (Lepidoptera:Nuctuidae) [59]. The extracts from *Salvia keerlii* and *Salvia ballotiflora* were shown to have modest insecticidal action (LV50= 1527 and 1685 µg mL^−1^, respectively. On the other hand, the extract of *S. ballotiflora* increased the larval and pupal stages by 5.2 and 2.9 days, respectively and caused a decrease in the pupal weight by 13.2%. Furthermore, *Salvia microphylla* showed insecticidal activity against FAW (LC_50_ = 919 ppm) [59]. The bioactivity of the essential oil of *S*. *ballotiflora* at 1000, 600, 400, 120 and 80 µg mL^−1^ led to reduced viability of larva which was 0%, 5%, 10%, 10%, and 20%, respectively [59]. They also reported extended duration of the larval stage by 30.5, 8.0, 5.5 and 5.5 days at 600, 400, 120 and 80 µg mL^−1^ compared with the control. The pupation period was extended by 1.6 days at 400 µg mL^−1^. Moreover, the reduction in weight of the pupae decreased by 52%, 39%, 29% and 29% at 600, 400, 120 and 80 µg mL^−1^, respectively, in relation to the control. 

*S. microphylla* contains palmitic acid, oleic acid and Y-sitosterol which have been associated with its activities against FAW [40]. Furthermore, they pointed out that there was a possibility to use of *Senecio salignus* and *Salvia microphylla* extracts for controlling FAW as they produce bioactive compounds that are antifeedants [91]. Salvia species are abundant in Africa including the South American exotic species *Salvia suaveolens* thus this species may be worthy of investigation to identify similar activities.

FAW eggs died at a rate of 97.7% one day after being exposed to extracts of *Lychnophora ericoides* and *Trichogonia villosa* [39]. Thus only 2.3% of the eggs hatched being a very low percentage to sustain populations that can cause damage.

Citrus-derived limonoids have been implicated in reduced feeding activity in insect pests. They include limonin, nomlin and abacunone and their semisynthetic products. Limonoids from *Citrus limon* have exhibited similar effects on FAW [83]. Citrus crops are also grown widely in Africa so further work on by-products of the peel from the fruit processing sector may provide opportunities for bioactive plant compounds in Africa. 

The biological activity of extracts from various plant parts of wild and in-vitro plants of *Piper tuberculatum* on the 3rd instars of FAW in Brazil have been studied [78]. The dichloromethane (DCM): methanol (2:1) and ethanol extracts of leaves and stems and boiling water extracts of leaves, stems and spikes of *P*. *tuberculatum* showed no effects on FAW 3^rd^ instars across the dosage. However, the DCM: methanol (2:1) and ethanol extracts of mature spikes from wild and DCM: methanol (2:1) extract of in vitro plants were reported to have exhibited potential insecticidal activity on the 3^rd^ instars of FAW. This result suggests that there is a potential for direct use of *P. tuberculation* mature spike of EtOH extracts that would allow farmers to utilize their locally brewed alcoholic drinks as extraction solvents. It would also mean that using in vitro techniques, the respective bioactive compounds can be biologically synthesized in large quantities using in-vitro cell suspension cultures [92]. This may require adequate and well-equipped laboratories most of which are out of reach for the farming support and commercial systems in Africa. *P. tuberculatum* has palmitic and oleic acids which could be responsible for the reduced viability of the larvae at 33.3% and 48.5%, respectively with a concentration of 1600 ppm. 

The main components identified in *Carica papaya* seed were oleic acid (45.97%), palmitic acid (24.1%) and stearic acid (8.52%) [44]. When evaluated against FAW the viability of the larvae was reduced to 33.3% for oleic acid, 48.5% for palmitic acid and 62.5% for stearic acid at 1600 ppm. Single fatty acids in *C*. *papaya* possessed greater potential to kill the insect pest compared to the chloroform extract. Amongst the three, palmitic acid was the most active.

A high mortality of FAW was reported with extracts of Jatropha curcas, Militia ferruginea, Phytolacca dodecandra, Scinus molle, Melia abyssinica, Nicotiana tabacum, Lantana camara, Chenopodium ambroides, Azadirachta indica and Jatropha gossypifolia [26]. This is the first report where these plant species were evaluated against FAW in Africa-Ethiopia. Similar activities were reported for A. indica and N. tabacum against FAW supporting these earlier findings [23]. 

The neem tree, *A. indica* can control many pest species including FAW [24,70,93,94]. The deleterious properties of neem oils and extracts on pests are associated with the content of limonoids like azadirachtin which is a highly complex and effective molecule [69]. Azadirachtin, is freely decomposable, selective, non-mutagenic causing minimal harm to mammals and the environment and could present an excellent option for controlling FAW [67]. For example, egg laying by female FAW was about 50% lower on the neem treated than on untreated cloth [67]. However, this substance has limitations such as being highly costly, it cannot be synthesized chemically and has to be purified using expensive and sophisticated methods. It can be produced from large quantities of seasonally available seeds [94] so may not be so well suited to small holders in Africa. The main components occur in the seeds and even for a “low-tech” processing method require considerable effort to extract them. One additional problem with the use of neem is that the main active components including the various azadirachtin related structures are highly UV labile so may low residual effects in the field [95]. There is no standardization and control of quality in neem-based preparations manufactured in Brazil an indicator that it may not possible to reproduce the desired effects of the insecticide [96]. To increase effectiveness, controlled-release preparations of insecticides by polymeric encapsulation [97,98] has been done. Encapsulation of neem oil and extracts into films or polymeric walls shelters the active component and permits controlled release stopping the loss of unstable compounds and increasing their stability in the environment [95]. Although again this approach may be beyond the needs and scope of smallholders but illustrates technologies in development to improve persistence in the field for botanicals. 

In another study neem seed cake extract was more active (LC_50_ = 0.13%) than leaf extract (LC_50_ = 0.25%) [73]. This was because of the higher amounts of azadirachtin the most effective of the toxic tetranortriterpenoids, because 90% of azadirachtin is more intense in the neem cake after pressing the seeds [74]. Farmers often use Neem leaves when seed is unavailable. However, the concentration of the active constituents is very low in leaves such that it has low and potentially no efficacy so promoting the use of Neem leaves should be discouraged as poor efficacy may negatively influence farmer opinions about the value of plants as alternatives to synthetics. Additionally, the bioassay indicated a static effect on the growth of FAW caterpillars, as most of them exhibited their exuviae in the terminal part of the body, incompletely releasing them as expressed by [69] as it limits the ability of the insects to feed by affecting the physiological functioning of ecdysis and in cellular processes, eventually causing insect death. This process takes some time and that is why comparatively, there is low larval mortality and high pupal mortality [99].

*Zea diploperennis* was evaluated against FAW and indicated that methanol extract and residual fiber of the plant adversely influenced the size of pupae. The aqueous extract caused 100% of larval cumulative mortality [81]. 

An extract was obtained from the roots and aerial parts of *M. geometrizans* using methanol as a solvent. Its components were peniocerol, macdougallin and chichipegenin and the mixtures of peniocerol and macdougallin. They all exhibited insect growth regulatory and insect killing activities against FAW [100].

## 3. Future Prospects

The plant species reviewed above provide an illustration of the extent of work undertaken to identify new pest management options from plants. These plants have been shown to have biological activity against FAW through various modes of action If these initial indications of activities are to be translated to the African context then not only are the bioactivity of extracts in the laboratory required but also the chemistry of these activities needs to be determined and the materials tested in field conditions using tailored approaches to extraction that are appropriate for small scale farmers. 

The plants reviewed had a variety of modes of action in controlling FAW including induction of low feeding ratio through the action of oleic acid (74.2%) and palmitic acid (7.16%) [44], repellent effects, severe toxicity, non-preference and knockdown effects by linalool from *Ocimum basilicum*. Citronella oil changes the chemical profile of FAW larvae, affecting reproductive and cell physiological parameters causing reduced reproduction and sometimes reproductive failure. It is also associated with changed epithelium that has cytoplasmic projections, columnar cell extrusion, pyknotic nuclei and increased periodic acid-schiff positive granules. Citronella oil caused morphological changes of the midgut and reduction of stored resources in the fat body, which may adversely affect insect reproduction and survival. It has been further reported that reduced feeding after ingestion of *Melia azedarach* caused starvation. This in addition to ingestion of toxic substances from *M. azedarach* [60]. Leaf extracts at vegetative and reproductive phase of Poinsettia (*Euphorbia pulcherrima*) increased larval period, reduced the weight of larvae and pupae egg viability [54]. The methanolic extracts of *Copaifera langsdorffii* leaves, bark of fruits and fruit peels resulted in low egg viability, reduction in larval growth, prolonged period of development, increased mortality, lowered fertility and fecundity of adults, abnormalities in the aeropylar and micropylar regions, increased excretion of protein in the insect feces and invitation of trypsin activity [79]. 

There is adequate evidence as indicated by the research findings presented in this review, that there are numerous opportunities for the use of botanical extracts in the management of FAW. However, exploitation of these opportunities is limited because the potential for use may face challenges attributed to the following:

1. Despite there being numerous plant products many are unstable upon application because they are UV labile. This means they may need more frequent application incurring greater costs in time. However, as they are non-persistent, they are potentially less damaging to the environment particularly non-target insects [17,18,99,101].
2. African smallholder farmers are not economically endowed to buy the botanical pesticides as has been the case for other farm inputs [102,103]. This therefore means that farmers will be encouraged to self-harvest these plant materials [104,105] and use them as crudely produced products as reported earlier [21,22].
3. There are different modes of action, which are determined by the stage of growth of both maize and the FAW raising the issue of exposure period, effectiveness, mode of application and method of extraction. However different modes of action could help to reduce the build-up of resistance in the pest where used in combination.
4. The opportunities maybe limited in scope where the products are not standardized for reproducibility and scale-up and this will require uniformity of the chemistry for the plant material and they likely need propagation [87,106]. More so, surprisingly few have been evaluated under field conditions [26]. This is a major oversight of the work as it means there is little evidence that any of the biological activities translate to a real-world setting. Field evaluations provide options to engage with farmers and determine effects on yield and determine non-target effects as undertaken recently in the African context [17,85,101].
5. Some of the plant materials tested may not be available for use by the farmers, for example the use of citrus seeds at farm level may not be attainable because it may not be feasible in terms of availability of the seeds. However, there may be good opportunities for propagation, and this would likely overcome some of the challenges of chemical variation across plant populations and provide consistency, which may otherwise be lacking when plants are harvested from the wild [106].
6. All the studies conducted failed to include economic viability for the tested plant extracts. This is closely linked to sustainable availability of the plant materials which is key driver for farmer adoption. Most of the plant materials used in these studies were wild harvested and this may not be self-sustaining unless efforts are made to commercialize the promising plant products or at least determine the economic viability of their use compared to alternatives including the use of synthetics [18,85].

## 4. Recommended Research Areas for Further Studies

1. Evaluate different extraction methods with the aim of documenting the most appropriate for adoption by small-scale maize farmers.
2. Investigate modes of action of different products based on part used, pure compounds and mixtures across the different growth stages of maize plant and FAW.
3. Conduct field evaluations of these plants and potentially determine any benefits of combining materials that could deliver different mechanisms of activity to address issues of insect resistance.
4. Investigate standardization to increase the scope of reproducibility and adoption especially through propagation.
5. Conduct research on approaches for upscaling, commercialization and sustainability of the botanical extracts and products
6. Testing activity of pure compounds from extracts to determine which components are active allowing the evaluation of variability across materials and improving methods for optimizing extraction and [87,107,108].

## Figures and Tables

**Table 1:** Plant species that have been evaluated for their activity against Fall Armyworm (FAW) and potential for use in its management.

Family	Plant Species	Action	Refs
Amaranthaceae	*Dysphania (*syn*. Chenopodium) ambrosioides* L. Mosyakin & Clemants	Mortality, decreased pupal weight	[26,27]
Anacardiaceae	*Schinus molle* L.	High mortality	[26]
Annonaceae	*Annona squamosa* L.	Decreased pupa weight, increased larval mortality	[33]
Apiaceae	*Foeniculum vulgare* Mill.	Sublethal effects	[34]
Apocynaceae	*Calotropis procera* (Aiton) W.T. Aiton	Decreased pupa weight, increased larval mortality	[33]
Asparagaceae	*Yucca periculosa* Baker	Growth regulating activity, increased developmental period, insecticidal activity, reduced pupation survival, reduced insect growth	[35]
Asteraceae	*Ageratum conyzoides* L.	Insecticidal (70% mortality)	[24]
Asteraceae	*Baccharis genistelloides* (Lam.) Pers.	Mortality	[36]
Asteraceae	*Artemisia verlotiorum* Lamotte	Mortality	[36]
Asteraceae	*Roldana barba-johannis* (DC.) H. Rob. & Brettell	Insecticidal	[37]
Asteraceae	*Gutierrezia microcephala* DC. A. Gray	Longer time for pupation and emergence of adults, severe toxicity against adults, insect growth inhibitory activity	[38]
Asteraceae	*Lychnophora ericoides* Mart.	Egg mortality	[39]
Asteraceae	*Trichogonia villosa* (Spreng.) Sch. Bip. Ex Baker	Egg mortality	[39]
Asteraceae	*Lychnophora ramosissima* Gardner	Larvicidal	[39]
Asteraceae	*Vernonia holosenicea* Mart. Ex DC.) L.	87% mortality	[39]
Asteraceae	*Senecio salignus* DC.	Antifeedant, insecticidal, juvenomimetric activity	[40]
Asteraceae	*Tagetes erecta* L.	Antifeedant effect causing 50% reduction of larval weight, 40%–80% pupal mortality, 48%–72% larval mortality	[41]
Cactaceae	*Myrtillocactus geometrizans* Mart. Ex Pfeiff	Insect growth regulating, larvicidal, delayed pupation	[41]
Cardiopteridaceae	*Cymbopogon winterrianus* Jowitt.	Alters biochemical profile of larvae, diminished reproduction, reproductive failure	[42,43]
Caricaceae	*Carica papaya* L.	90% mortality	[44,45,46]
Celastraceae	*Maytenus disticha* (Hook. F) Urb.	Insecticidal activity	[47]
Celastraceae	*M. boaria* (Molina)	Insecticidal activity	[47]
Convovulaceae	*Ipomoea murucoides* Roem. And Schult	46.16% mortality, reduced larval weight increased pupation time	[48,49]
Euphorbiaceae	*Ricinus communis* L.	Insecticidal and insectistatic, larvicidal, growth inhibition	[50,51]
Euphorbiaceae	*Jatropha curcas* L.	High mortality	[26]
Euphorbiaceae	*Jatropha gossypiifolia* L.	Antifeedant to larva, synergistic with pesticide	[52]
Euphorbiaceae	*Euphorbia pulcherrima* Willd. Ex Klotzsch	58.5% mortality, reduced larva and pupae weight, increased larva period, reduced egg viability	[53,54]
Leguminosae	*Copaifera langsdorffii* Desf.	Low fertility and fecundity, low viability of eggs, larval growth reduction, inhibited trypsin activity, egg abnormalities	[55]
Leguminosae	*Militia ferruginea* Hochst.	High mortality	[26]
Lamiaceae	*Ocimum basilicum* L.	Toxicity, non-preference, knockdown	[23,56,57]
Lamiaceae	*Ocimum gratissimum* L.	Sublethal effects	[58]
Lamiaceae	*Salvia keerlii* Benth.	Insecticidal	[59]
Lamiaceae	*Salvia ballotiflora* Benth.	Insecticidal, insectistatic, increased larval and pupal duration, reduced pupa weight	[59,60]
Lamiaceae	*Salvia connivens* Epling	Insecticidal, insectistatic	[59]
Lamiaceae	*Salvia microphylla* Kunth	Antifeedant, insecticidal, juvenomimetric activity	[40]
Malvaceae	*Malva sylvestris* L.	Mortality	[36]
Meliaceae	*Melia azedarach* L.	Reduced larval feeding, reduced larval growth, synergistic with pesticide	[52,61]
Meliaceae	*Trichilia pallens* C. de Candolle	Mortality	[62,63]
Meliaceae	*Trichilia pallida* Sw.	Mortality	[63]
Meliaceae	*Cedrela salvadorensis* Standl.	Larval mortality, growth reduction, inhibited larval growth, reduced pupal weights and adult emergence	[64]
Meliaceae	*Cedrela dugessi* S. Watson	Larval mortality, growth reduction, inhibited larval growth, reduced pupal weights and adult emergence	[64]
Meliaceae	*Melia abyssinica*	High mortality	[26]
Meliaceae	*Trichilia pallida* Sw.	No egg deformities	[65]
Meliaceae	*Azadirachta indica* Juss.	Reduced insect growth, increased development period, mortality, low egg laying, antifeedant activity, growth regulating activity, mortality, larvicidal	[24,26,65,66,67,68,69,70,71,72,73,74,75]
Monimiaceae	*Peumus boldus* Molina	75% mortality	[76]
Moringaceae	*Moringa oleifera* Lam.	Low feeding ratio, (antifeedant activity) mortality	[77]
Myrtaceae	*Eucalyptus citriodora* Hook	Growth regulating activity	[29]
Myrtaceae	*Eucalyptus staigeriana* F. Muell. Ex Bailey	Sublethal effects	[34]
Myrtaceae	*Eucalyptus globulus* Labill.	High mortality	[26]
Myrtaceae	*Siphoneugena densiflora* Berg	100% larval mortality	[78]
Petiveriaceae	*Petiveria alliacea* L.	Mortality	[36]
Phytolaccaceae	*Phytolacca dodecandra* L’Herit.	High mortality	[26]
Piperaceae	*Piper tuberculatum* Jacq.	Insecticidal	[79]
Piperaceae	*Piper hispidinervum* C. DC.	Affects spermatogenis and egg laying	[79]
Poaceae	*Cymbopogon citratus* (DC.) Stapf	Mortality	[36]
Poaceae	*Cymbopogon flexuosus* Steud.	Toxic, insecticidal activity	[80]
Poaceae	*Cymbopogon nardus* L.	Decreased pupa weight, increased larval mortality	[33]
Poaceae	*Zea diploperennis* L.	High larval survival	[81]
Rhamnaceae	*Zizyphus joazeiro* Mart.	Decreased pupa weight, increased larval mortality	[33]
Rubiaceae	*Morinda citrifolia* L.	Decreased pupa weight, increased larval mortality	[33]
Rubiaceae	*Psychotria goyazensis* Mull. Arg.	Reduced hatching rate, Egg mortality	[82]
Rutaceae	*Ruta graveolens* L.	Mortality	[36]
Rutaceae	*Citrus limon* L.	Antifeedant	[83]
Sapindaceae	*Magonia pubescens* A. St.-Hil.	Decreased pupa weight, increased larval mortality	[33]
Sapindaceae	*Talisia esculenta* Rsdlk.	Mortality	[61]
Sapindaceae	*Sapindus saponaria* L.	Mortality	[61]
Solanaceae	*Nicotiana tabacum* L.	High mortality	[23,26]
Verbenaceae	*Lantana camara* L.	High mortality	[26]
Verbenaceae	*Vitex polygama*	High mortality	[84]
Zingiberaceae	*Zingiber officinale* L.	Mortality	[36]
